# A Case of Type 2 Diabetes Mellitus Revealed Transient Positivity of Glutamic Acid Decarboxylase (GAD) Antibodies Following Immunoglobulin Administrations

**DOI:** 10.7759/cureus.66485

**Published:** 2024-08-09

**Authors:** Muneo Kawasumi, Shogo Ishii, Shota Uchida, Takeo Shishido

**Affiliations:** 1 Department of Endocrinology and Diabetology, Hiroshima City North Medical Center Asa Citizens Hospital, Hiroshima, JPN; 2 Department of Neurology, Hiroshima City North Medical Center Asa Citizens Hospital, Hiroshima, JPN

**Keywords:** guillain-barré syndrome, slowly progressive type 1 diabetes mellitus (spiddm), type 2 diabetes mellitus (t2dm), immunoglobulin, glutamic acid decarboxylase antibody (gad antibody)

## Abstract

Glutamic acid decarboxylase (GAD) antibodies are frequently measured in diabetes care as islet-associated autoantibodies that are useful in the diagnosis of type 1 diabetes. However, GAD antibodies derived from other persons may contaminate immunoglobulin preparations, and there have been cases of transiently positive GAD antibodies after intravenous immunoglobulin (IVIg) in patients who were originally negative for GAD antibodies. Clinicians may be unaware of such contamination and misdiagnose some cases as type 1 instead of type 2 diabetes mellitus based on positivity for GAD antibodies. Herein, we present a case of type 2 diabetes mellitus that revealed transiently positive GAD antibodies following immunoglobulin administrations. A 68-year-old woman with a medical history of diabetes mellitus was admitted to our hospital for the treatment of Guillain-Barré syndrome, and IVIg was started on the day of admission. Blood tests on admission revealed negative for GAD antibodies but showed weak positivity on day one after IVIg. Afterward, GAD antibodies turned negative on day 72. Immunoglobulin preparations were revealed to have a high concentration of GAD antibodies. Based on changes in GAD antibody titers and all negativity for anti-insulinoma-associated antigen-2 (IA-2), insulin, and zinc transporter 8 (ZnT8) antibodies, the patient was diagnosed with type 2 diabetes mellitus rather than slowly progressive type 1 diabetes mellitus (SPIDDM). This case demonstrates that it is important for the medical clinician to be aware of the possible presence of GAD antibodies in immunoglobulin preparations and to measure antibody titers before and after their use for diagnosing the type of diabetes mellitus.

## Introduction

There are four main types of diabetes mellitus: type 1, type 2, diabetes mellitus associated with genetic abnormalities or other diseases such as liver and pancreatic disease, and gestational diabetes mellitus. The destruction of pancreatic beta cells leads to type 1 diabetes, resulting in decreased insulin secretion and deficiency. Etiology is thought to be an autoimmune reaction against pancreatic beta cells, and islet-associated autoantibodies are often detected in patient serum. Glutamic acid decarboxylase (GAD) antibodies are widely known as islet-associated autoantibodies that are useful in the diagnosis and prediction of type 1 diabetes [1-3].

Commercially available intravenous immunoglobulin (IVIg) preparations are human blood products used to treat autoimmune diseases, such as Guillain-Barré syndrome. Scattered reports have revealed transient positive GAD antibodies due to the use of immunoglobulin preparations [4-6]. In the case of the positive for GAD antibodies after using immunoglobulin preparations, the patient with type 2 diabetes mellitus may be misdiagnosed as type 1 diabetes mellitus. Therefore, the general clinician should confirm the history of treatment with immunoglobulin when diagnosing the type of diabetes mellitus.

We herein report a case of a 68-year-old woman with type 2 diabetes mellitus that revealed transient positivity for GAD antibodies following immunoglobulin administrations.

## Case presentation

A 68-year-old woman was transferred to our neurological department by ambulance due to limb weakness and numbness that had worsened over the previous three days. She presented with common cold symptoms two weeks prior to transport. She had a medical history of breast cancer, diabetes mellitus, and hypercholesterolemia, and she was taking mitiglinide 30 mg/day, voglibose 0.6 mg/day, canagliflozin 100 mg/day, vildagliptin 100 mg/day, metformin 1,000 mg/day, and atorvastatin 5 mg/day. The duration of diabetes mellitus was 17 years, but the detailed treatment history before being transported to our hospital was unknown. A family history indicated diabetes in her parents and sisters.

When the patient arrived at our hospital, her Glasgow Coma Scale (GCS) was 15/15, blood pressure was 144/99 mmHg, heart rate was 125 beats/minute with sinus tachycardia, respiratory rate was 14 breaths/minute, blood oxygen saturation was 97% at room air measured by a pulse oximeter, and body temperature was 37.0 ℃. The patient weighed 50.5 kg and had a BMI of 22.1 kg/m^2^. Diabetic peripheral neuropathy was difficult to assess, but stage 2 of diabetic nephropathy and simple retinopathy in both eyes were observed. The neurological examinations revealed dysphagia, mild dysarthria, weakness of proximal muscles of both upper and lower limbs, paresthesia at the ends of the extremities, mild impaired vibration sense of both lower limbs, and loss of patellar and Achilles tendon reflexes. Laboratory investigations showed leukocytosis, neutrophilia with white blood cell (WBC) count of 10,030/µL (reference range: 3,300-8,600/µL), segmented neutrophil count of 7,890/µL (reference range: 1,500-6,000/µL), and elevated C-reactive protein levels of 0.533 mg/dL (reference range: 0.000-0.140 mg/dL). Creatine phosphokinase (CPK) levels were normal (57 U/L, reference range: 41-153 U/L). Her glycated hemoglobin (HbA1c) level was high (7.5%, reference range: 4.9-6.0%), and the test for glutamic acid decarboxylase (GAD) antibodies (reference range: < 0.5 U/mL) was negative. The cerebrospinal fluid analysis revealed protein-cytological dissociation with cells of 3.0/mm^3^ (reference range: 0-5/mm^3^) and total proteins of 49.0 mg/dL (reference range: 10-40 mg/dL). Chest and abdominal computed tomography (CT) was normal. Magnetic resonance imaging (MRI) of the spine revealed no abnormalities in the vertebral bodies, discs, and vertebral alignment. There was no stenosis in the spinal canal and no abnormal high signal in the spinal cord. A nerve conduction test showed a mild prolongation of distal latency of the motor nerves in the upper right extremity and a mild decrease of motor nerve conduction velocity in the lower right extremity. In addition, there was a poor derivation of sensory nerve action potential amplitude (SNAP) in the upper right extremity, while the peroneal nerves were normal. These findings suggested a demyelinating neuropathy. A diagnosis of Guillain-Barre syndrome (GBS) was confirmed based on the above findings, and intravenous immunoglobulin (IVIg) was started (20 g intravenously per day for five consecutive days).

Laboratory investigation on day one after admission revealed euglycemic ketoacidosis (EKA) with a plasma glucose level of 177 mg/dL, arterial blood pH of 7.179, bicarbonate level of 8.5 mmol/L, and 3+ urinary ketone bodies. The patient had been taking canagliflozin 100 mg/day until the day before being transported to our hospital. The total ketone body level was 10,500 μmol/L (reference range: 26-122 μmol/L), the acetoacetic acid level was 2,820 μmol/L (reference range: 13-69 μmol/L), and the 3-hydroxybutyric acid level was 7,730 μmol/L (reference range: 0.0-76 μmol/L). Serum C-peptide levels were within the normal range (0.8 ng/mL, reference range: 0.8-2.5 ng/mL), but the C-peptide index (CPI) was low (0.5). The urinary C-peptide reactivity was decreased (33.4 μg/day). This was a case of diabetes with decreased endogenous insulin secretion. Autoimmune tests were performed, with negative anti-insulinoma-associated antigen-2 (IA-2) antibodies and anti-insulin antibodies. The retest for GAD antibodies was performed to exclude type 1 diabetes due to decreased insulin secretion ability. GAD antibodies were positive (22.1 U/mL) on day one, although negative on admission. The test for zinc transporter 8 (ZnT8) antibodies was not performed at this time.

After being admitted to the high care unit with the diagnosis of EKA complicated by GBS, the patient was given intravenous insulin infusion (1 unit/hour for Humulin RR) and intravenous fluid infusion (4.2 g/hour for glucose). Arterial blood pH improved to 7.359 after 14 hours of treatment, and we considered it to be the recovery from EKA. Subsequently, the intravenous insulin infusion was discontinued.

**Figure 1:**
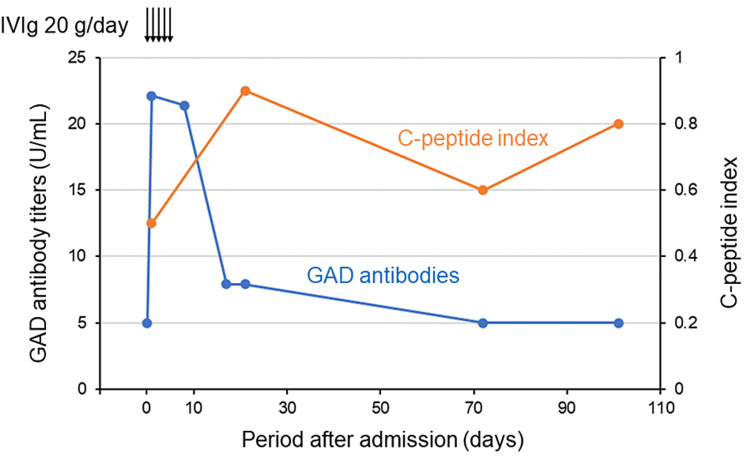
Patient’s clinical course The GAD antibody titers less than 5.0 U/mL are denoted as 5.0 U/mL. IVIg: intravenous immunoglobulin; GAD: glutamic acid decarboxylase

Since the endogenous process where the negative GAD antibodies turned positive 31.5 hours later was considered unnatural, the involvement of immunoglobulin preparations was suspected. All immunoglobulin preparations used in our patient were from the same lot (lot number 538976, Takeda Pharmaceutical Company Limited) and had a high concentration of GAD antibodies, as tested with enzyme-linked immunosorbent assay (ELISA) (71.2 U/mL). Immunoglobulin preparations were administered for five days from the day of admission, and a second dose was not administered because of the improvement in neurological symptoms. On day eight, after the initiation of IVIg, GAD antibody titers remained unchanged at 21.4 U/mL, but they decreased to 7.9 U/mL nine days later (Figure 1). On day 21, when our patient was transferred to another hospital for rehabilitation, GAD antibody titers remained at 7.9 U/mL. Based on the clinical course, our patient was considered unlikely to have type 1 diabetes. Diabetic medication adjustment resulted in a blood Glucose transition stabilized by glimepiride 1mg/day and vildagliptin 100 mg/day at this time. During follow-up at our outpatient clinic on day 72 after the initiation of IVIg, GAD antibodies were confirmed to be negative at less than 5.0 U/mL. A blood test on the same day showed HbA1c of 7.3%, serum C-peptide levels of 0.9 ng/mL, and CPI of 0.6. On day 101, GAD antibodies were still negative, while HbA1c and serum C-peptide levels were elevated to 7.6% and 1.2 ng/mL, respectively. CPI remained low at 0.8, indicating reduced insulin secretory capacity. Therefore, glimepiride was discontinued, and diabetes treatment was converted to vildagliptin 100 mg/day and insulin glargine 3 units/day. Four months after admission, zinc transporter eight antibodies (reference range: < 15.0 U/mL) were negative. Given the trend in GAD antibody titers and other negative islet-associated autoantibodies, in addition to the absence of insulin secretory deficiency, our patient was diagnosed with type 2 diabetes, not type 1 diabetes mellitus, such as slowly progressive insulin-dependent diabetes mellitus (SPIDDM).

## Discussion

We treated a case of type 2 diabetes mellitus that revealed transient positivity of GAD antibodies following immunoglobulin administrations. To the best of our knowledge, there are no reported cases of type 1 diabetes immediately after the onset of Guillain-Barré syndrome. The prevalence of GAD antibody positivity in patients with type 1 diabetes was reported to be 74.2% for less than one year, 63.0% for one to three years, 74.3% for four to nine years, and 66.7% for more than 10 years after the onset of the disease [7]. Regardless of the duration of type 1 diabetes, the prevalence of GAD antibody positivity was high, suggesting a prolonged positive period. On the other hand, in diabetic patients with transient positivity of GAD antibodies after administration of immunoglobulin preparations, GAD antibodies turned positive on day three at the earliest and negative on day 127 at the longest [5,6]. Many immunoglobulin preparations used contained GAD antibodies, although there was some variation in GAD antibody titers between each lot. The GAD antibody titers in the patient's serum after administration of the immunoglobulin preparations were 2.1-2.9 U/mL, as measured by radioimmunoassay (RIA), and 32.4-33.7 U/mL, as measured by enzyme-linked immunosorbent assay (ELISA), indicating a weak positive result. In this case, GAD antibodies, which were negative at the time of initial diagnosis, turned positive on day one after administration of the immunoglobulin and negative on day 72. All immunoglobulin preparations used were in a single lot, and GAD antibodies with a concentration of 71.2 U/mL were detected in the same lot. The GAD antibody titers in our patient's serum were weakly positive at 22.1 U/mL, according to ELISA. We could not completely rule out the possibility that the initial test for GAD antibodies was a false negative and that the patient had SPIDDM. However, the negative for GAD antibodies on admission was likely to be correct based on the above findings. The positive GAD antibody response could have been a direct effect of contamination of GAD antibodies from other persons in the immunoglobulin preparations and an indirect effect of endogenous GAD antibody production triggered by the administration of the immunoglobulin preparations. GAD antibodies derived from other persons may be contaminated in immunoglobulin preparations. Dimitriadou et al. reported that ELISA detected GAD antibodies in 15 out of five different 16 immunoglobulin preparations [8]. In addition, there have been reports of transient positive changes in GAD antibodies after the administration of immunoglobulin preparations to patients with neurological disease without diabetes mellitus [9,10]. The immunoglobulin preparations used in this case were manufactured in Japan from donated blood plasma collected by the Japanese Red Cross Society. According to the Japanese blood donation standards, blood donations from people taking diabetes medications, including insulin, oral hypoglycemic drugs, antibody drugs, and immunosuppressive drugs, are not allowed. Blood from patients with GAD antibodies, including type 1 diabetes and stiff-person syndrome, is unlikely to be contaminated in blood donation products due to strict blood donation standards. GAD antibodies appear in the blood several years before the onset of type 1 diabetes [1]. A report from Norway showed a GAD antibody positivity rate of 1.7% in healthy people without diabetes, with the highest antibody titers being 30 times the cutoff value [11]. Therefore, the GAD antibodies in the immunoglobulin preparations were considered to be derived from the blood of healthy asymptomatic people.

Immunoglobulin preparations differentiate B cells into IgG-producing cells in an in vitro culture system [12]. In addition, Brem et al. revealed that the administration of immunoglobulin preparations to patients with Guillain-Barré syndrome contributed to an increase in peripheral blood plasmablasts and subsequent endogenous IgG production [13]. As far as we could find, no papers reported the detection of endogenous GAD antibodies after administering immunoglobulin preparations not contaminated with GAD antibodies of others to GAD antibody-negative patients. However, in light of the above, the possibility that the administration of immunoglobulin preparations triggered endogenous GAD antibody production could not be ruled out.

Following the diagnostic criteria for SPIDDM published by the Japan Diabetes Society (2023), this case was classified as slowly progressive type 1 diabetes mellitus (probable) [2]. This patient was diagnosed with diabetes mellitus at our hospital 17 years ago based on blood glucose levels of 309 mg/dL and HbA1c measurement of 14.8%. There was no ketosis at diagnosis, but insulin therapy was required because of the preoperative condition for uterine fibroids. At that time, GAD antibodies were not measured. When the patient was brought to our hospital for treatment of Guillain-Barré syndrome, she tested negative for GAD antibodies but tested positive on the first day after administration of the immunoglobulin preparations.

However, GAD antibodies showed negative results on day 72. Although insulin secretory capacity decreased, IA-2, insulin, and ZnT8 antibodies were all negative. Given the above findings, the patient was considered to have type 2 diabetes mellitus rather than SPIDDM. Without data on GAD antibody titers after administration of the immunoglobulin preparations, the patient would have been treated as SPIDDM and disadvantaged physically, mentally, and financially. Treatment options in the case of SPIDDM are limited to dipeptidyl peptidase-4 (DPP-4) inhibitors, biguanides, insulin, and glucagon-like peptide-1 (GLP-1) receptor agonists, as these agents have evidence of benefit [14]. The introduction of intensive insulin therapy may be considered if glycemic control goals are not achieved through the combination of these agents. Although our patient was started on insulin therapy with glargine due to decreased endogenous insulin secretion, the diagnosis of type 2 diabetes mellitus provided her with more treatment options than in the case of SPIDDM. The time to introduction of intensive insulin therapy was expected to be longer. When immunoglobulin preparations are used in autoimmune diseases, such as Guillain-Barré syndrome, GAD antibody titers should be measured before and after administration.

## Conclusions

We reported a case of type 2 diabetes mellitus that revealed transiently positive GAD antibodies following immunoglobulin administrations. This report highlights that medical clinicians are crucial to be aware of the possible presence of GAD antibodies in immunoglobulin preparations and to measure patients’ antibody titers before and after their use for diagnosing the type of diabetes mellitus. If GAD antibodies become positive due to the administration of the immunoglobulin preparations, the patient could be misdiagnosed with SPIDDM and be disadvantaged physically, mentally, and financially in the future. When GAD antibody positivity is observed after immunoglobulin administrations, GAD antibody titers should be followed since GAD antibodies derived from others turn negative after 127 days at the longest.
